# Dealing with the death of a long term patient; what is the impact and how do podiatrists cope?

**DOI:** 10.1186/s13047-017-0219-0

**Published:** 2017-08-08

**Authors:** Kristy Robson, Cylie M Williams

**Affiliations:** 1 School of Community Health, Charles Sturt University, Albury, NSW 2640 Australia; 2 Department of Physiotherapy, Monash University, Frankston, VIC 3199 Australia; 3 Peninsula Health Allied Health, Frankston, VIC 3199 Australia

**Keywords:** Health workforce, Grief, Resilience

## Abstract

**Background:**

It is common for podiatrists and patients to develop long term professional relationships. Patient’s decline in health or death may impact a practitioner’s mental wellbeing. This research aimed to understand the impact of long term patient death on podiatrists and identify coping strategies.

**Method:**

Australian podiatrists were eligible to participate if they had been practicing longer than 5 years and experienced the death of a long term patient in the previous 12 months. Individual semi-structured interviews were conducted with podiatrists and were audio-recorded, transcribed verbatim and individually analysed to identify key themes. Interpretative phenomenological analysis was used to explore the perceptions of podiatrists on the personal and professional impact following the death of a long term patient.

**Results:**

Fifteen podiatrists (11 female) with a median of 15 (range 8–50) years’ experience participated.

Three major themes emerged: acknowledging connections, willing to share and listen, and creating support through starting the conversation. Participants indicated importance in recognition of the emotional influence of professional-patient relationships. They also discussed the importance of debriefing about death with the right person, which was most commonly colleagues. Participants talked about the emotional impact of death, suggesting the need for supporting discussion and resources, especially for new graduates.

**Conclusion:**

Death and dying can be an emotive topic and one which podiatrists may not be prepared for, yet likely to have to deal with throughout their career. These findings enable a better understanding of the impact of patient death and provide possible future directions for the profession to better support podiatrists in this area.

## Introduction

Professional support and coping with experiences surrounding patient death has traditionally thought to be a medical or nursing issue. Studies have investigated the impact of patient death on these groups of health care professionals [1, 2], their attitudes [3] and practices at time of death [2]. Medical and nursing professionals commonly have the most exposure to death of a patient through acute or chronic illness, and most often this is within an inpatient hospital setting.

There are a variety of factors that may result in different grief reactions from health professionals when faced with death. These may include the workplace setting or patient health status. Differences have been described between the levels of grief experience and reactions during an emergent health crisis of a new patient [4] versus the long term relationship between a health profession and their patient during chronic illness [1]. Health professional’s grief may also be dramatically impacted by the age of the patient [2].

Coping skills, breaking bad news to families and supporting colleagues through death are often developed and modelled throughout undergraduate degrees for health professionals [5] and in fellowship training programs for medical professionals [6]. Yet many health care professionals have described feeling inadequately prepared when encountering patient death [7]. Many health care organisations have responded to this with the development of supporting structures for both individuals and departments [2, 7]. However training and the impact of patient death appears relatively unspoken about in allied health.

Many podiatrists working in the public and private setting provide ongoing care for patients. In the acute system, they may establish an intermittent relationship with a patient who has a wound and may re-present over the course of years for treatment. In the private setting, there may be short term care during an episode of pain, long term care such as routine nail care for older patients or as children grows with a chronic medical health condition impacting their gait. Anecdotally, podiatrists have shared the impact of an unexpected or expected patient death within work places and when talking to students, however this has never been formally investigated. Some podiatrists have also described burnout as a result of primarily working with the client group who typically present to podiatry services [8, 9]. The aim of this study was to explore the professional and personal impact on podiatrists following the death of a long term patient.

## Methods

This research project received approval from the Monash University Human Ethics Committee (MUHREC approval: 553)

### Study design

A qualitative research approach was chosen to explore the impact of the death of a long term patient on podiatrists and to identify common coping strategies. The use of a qualitative approach allows the focus to be on the participants’ experiences and knowledge related to the specific topic [10]. Adopting this approach enables an in-depth understanding of the topic through discussions or observations with the participants [11].

Phenomenology is a qualitative approach that explores human behaviour and the meaning people attribute to their experiences, particularly how they interpret life events in order to understand what it is like to be human [12]. The specific phenomenological approach chosen for this research was interpretative phenomenological analysis (IPA) due to its focus towards an individual’s interpretation of the meaning of everyday life experiences [11]. IPA was considered an appropriate method as the participants were asked to discuss the impact of the phenomenon, in this case how experiencing the death of a long term patient, impacted on their professional and personal interactions. We were particularly interested in exploring how practitioners dealt with this event on an individual level [13]. The interpretative nature of IPA, informed by the interpretation theory of hermeneutics, enabled the participant to reflect on their experience while the researcher interpreted the participant’s reflection in order to understand their unique experience [12]. Central to IPA is gathering and analysing data from a small number of purposively selected participants who have a common experience of a specific phenomenon. This enabled a detailed analysis of the phenomenon encompassing each participant’s experience through a process of idiography [12]. There is no specific rule determining the required numbers of participants within an IPA study, but rather sample size should be governed by the depth of analysis, the richness of data from individual participants and the design of the study [12, 14]. IPA studies have been published with participant numbers ranging from 1 to 30 participants [15]. As such, the intention of this study was to recruit 10 to 15 participants that met the inclusion criteria.

### Sampling

In line with the aims of the study, a purposive sampling approach was applied. Eligible podiatrists had registration with the Australian Health Practitioner Regulation Agency (AHPRA) and had experienced a death of a patient within the last the last 12 months who they had been treating for a minimum of 5 years. Furthermore, we wished to purposively sample podiatrists who had a broad range of reaction(s) to dealing with death of a long term patient. Following consent, participants completed the Brief Resilience Scale (BRS) and the Abbreviated Maslach Burnout Inventory (aMBI) to ascertain levels of distress and/or burnout. These tools are both valid and reliable measures of assessing resilience or stress recovery [13] and levels of emotional exhaustion, depersonalisation, and personal accomplishment [14, 15]. Burnout was indicative with scores of 27 in emotional exhaustion, greater than 10 in depersonalisation and less than 33 in personal accomplishment [15]. A score between 3.00–4.30 was indicative of normal resilience [16]. As such, these scores were used as a basis for excluding participants as it is well evidenced that health professionals suffering from high levels of distress and burnout have difficulty with empathy [17], optimism [18], mood regulation [18] and positive attitudes towards patient care [19]. Results indicative of extreme depersonalisation or hypersensitivity on these scales were used as exclusion criteria to ensure participants were not experiencing high levels of distress and burnout at the time of data collection which could have impacted on the study results.

### Participant recruitment

Participant recruitment was via advertisements through the Australian Podiatry Associations in all Australian states as well as social media avenues. Participants interested in being involved in the study were encouraged to contact the researchers directly via email or phone. At this point, they were screened for eligibility to be included in the study. Potential participants then completed the two questionnaires and these were scored before data collection was undertaken.

### Data collection

Individual semi-structured telephone interviews were undertaken with the participants. The use of telephone interviews in qualitative studies have shown to be a useful strategy when participants are geographically disperse [20]. There is also evidence supporting the positive aspect of telephone interview when discussing difficult topics. Telephone interviews can enable participants to talk more freely, because they remain in their own environment and it can be less confrontational when discussing sensitive topics [21, 22]. Within the context of IPA, a number of different of data collection approaches have been reported, these include in-depth interviews, analysis of participant diaries, focus groups sessions or participants observations [12; 11]. The underlying premise of data collection within IPA, regardless of the approach, is to ensure that participants are provided with an opportunity to discuss their experiences by speaking freely and reflectively [12]. Participant interviews were guided by an open-ended questioning approach, such as “tell me about your most recent experience of one of your long-term patients passing away?”. These questions were phrased to encourage conversation and to facilitate detailed answers. Where needed, additional probing questions, such as “how did this experience make you feel?” were also employed to encourage elaboration of initial responses [23]. Each participant was guided to talk about their experiences following the death of a long term patient, including the coping strategies they used and support they required. Interviews were audio-recorded, transcribed verbatim and then emailed to the participant for member checking prior to the commencement of data analysis to reduce bias and ensure validity.

### Data analysis

The IPA data analysis guidelines were used to guide analysis [12]. The process involved the in-depth analysis of one participant’s transcript prior to moving onto the next transcript to ensure that each participant’s unique experience was noted before identifying patterns across participants. This approach is congruent with the theoretical orientation of ideography within IPA methods, where in-depth and detailed analysis is undertaken to prevent the pre-mature interpretations of themes and generalisations during the data analysis process [12, 14].

The process of data analysis was divided into four steps: 1) Each individual transcript was read multiple times to create familiarity with the story; 2) Descriptive notes were made to summarise/highlight key and relevant points; 3) These notes were used to identify emergent themes unique to the individual participant, with similar themes eventually being grouped together; and 4) When the analysis of each participant transcript was completed, emergent themes were compared and contrasted between the participants’ transcripts to develop overarching super-ordinate themes.

### Trustworthiness

In order to demonstrate the authenticity of research findings to the lived experience of the participants, consideration of trustworthiness must be demonstrated [24]. Trustworthiness of this study was achieved through various means including, member checking, researcher triangulation, transferability and reflexivity. Following transcription, the interviewer read the interview to ensure it was reflective of the questions asked, then each participant reviewed their transcript before data analysis began through a process of member checking to provide participants the opportunity to clarify meaning and adjust responses [24]. Researcher triangulation was attained by both authors, who are skilled researchers, being involved in the data analysis phase to reduce the risk of individual researcher bias [25]. This was achieved by one researcher (CW) undertaking all of the semi-structured interviews with participants and then the second researcher (KR) undertaking the data analysis and developing the emergent and super-ordinate themes before being further reviewed by the first researcher (CW) to ensure authenticity with the participants lived experience. A detailed description of the particular participant group included in the study was provided to enable judgements of transferability of the study [24]. Reflexivity involves an acknowledgement that researchers can influence the research through personal biases [11]. Both researchers, who are registered Australian podiatrists, have had previous experiences of long term patient death. Transcription of interviews included mentions of when the interview was paused for the participant to gather composure, if there was participant laughter or crying. Throughout the data analysis process, the second researcher (KR) kept a reflective journal to document her thoughts understandings of the participant’s stories, which also incorporated non-verbal verbal elements of the interview such as pauses and emotional elements such as laughter or crying. This process enabled an opportunity to enhance the research findings [26].

## Results

There were 18 podiatrists that contacted the researchers to participate. As we intended to recruit 10–15 participants, 15 participants were initially screened for eligibility on a “first come first served” basis and were subsequently included in the first round of data collection and analysis basis. It was intended that the remaining three participants would be included should further data be required. Of the 15 podiatrists (11 female) who participated, with a median of 15 (range 8–50) years of podiatry practice. Participants worked across the private sector including private practice with or without residential aged care service provision or the public sector including community health and/or acute hospitals.

Table 1 presents the participants’ pseudonym, gender, years of practice, and type of practice at the time of interview. Participants worked across Victoria, South Australia, New South Wales, Tasmania and Queensland but in order to maintain confidentiality, this information was not linked with the pseudonyms. The mean (SD) BRS was 3.48 (0.94) and the mean (SD) scores from the aMBI were 5.5 (3.6) in emotional exhaustion, 1.9 (2.7) for depersonalisation and 16.5 (1.7) in personal accomplishment. No participants had scores indicative of burnout, two participants scored moderately on the emotional exhaustion questions.

**Table 1:** Study participants

Pseudonym	Gender	Years of practice	Practice setting
Brett	Male	12 years	Private
Kara	Female	12 years	Public
Lisa	Female	20 years	Private
Emma	Female	22 years	Private
Melissa	Female	8 years	Public
Kate	Female	14 years	Private
Anna	Female	8 years	Public
Louise	Female	20 years +	Public
Megan	Female	50 years	Private
Steve	Male	16 years	Private
Jessica	Female	10 years	Public/Private
Fran	Female	34 years	Private
James	Male	8 years	Private
Annabelle	Female	23 years	Private
Tim	Male	13 years	Public

After 15 interviews were conducted, no new themes emerged and sufficient perspective was gained to illuminate the participant’s perspective on the aims of the study [27]. As such data saturation was achieved and no more interviews with the remaining participants were undertaken. Each interview took between 20 and 60 min.

Table 2 presents the emergent themes from the analysis of participants’ transcripts, which enabled the development of the overarching super-ordinate themes. All participants spoke about the uniqueness of the podiatry profession where opportunities typically present for podiatrists to spend extended periods of time with patients on a regular basis. This invariably meant they learned a lot about the patient. Kara and Brett captured the essence of what the other participants described about the established relationship with their patients.

And you become a part of their day. I know that sounds very strange, but even in that half hour you learn a lot about a person and when it’s week on week, according to them you become a part of their family. And there’s really nothing that you don’t know about them. (Kara)

We’re incredibly unique as a profession. We spend more time with our patients than most other people, and it’s a very personal part of the body for a lot of people as well. (Brett)

The participants acknowledged the importance in recognising the emotional influence of professional-patient relationships. Some described being more profoundly affected than others following the death of a patient. They discussed the usefulness of debriefing about death with the right person, most commonly colleagues. Participants also talked about the emotional impact of death, suggesting the need for supporting discussion and resources, especially for new graduates. Three super-ordinate themes emerged through the analysis of the interviews that related to how practitioners dealt with this impact. These were: 1) Acknowledging connections; 2) Willing to share and listen; and 3) Creating support through starting the conversation.

**Table 2:** Emergent themes and Super-ordinate themes

Emergent themes	Super-ordinate themes
• Podiatrists being embedded in patient lives • Building of relationships • The ripple effect onto staff when someone dies • The additional stress of managing family members of the patient who has died • Emotionally invested due to the ongoing continuity of care • Often in a position to listen and understand a patients personal lives and belief/values • Links to your own personal life • Deep professional relationship not always recognised by others	*Acknowledging Connections*
• Need to find the right listener • Important to have a support mechanism • Debriefing with people who understand the context • Importance of being open to seeking to help • Understanding that everyone has their own process of grieving • Having a supportive workplace • Recognising the emotional impact • Devaluing the impact because it is work related	*Willing to share and listen*
• Increasing general discussion on the topic • Greater opportunity to have formal training on understanding the impact • Empowering practitioners with knowledge on how to support themselves • Increasing emotional awareness through effective strategies • Recognising the emotional impact within the podiatry progression • Better training for students	*Creating support through starting the conversation*

### Theme 1: Acknowledging connections

All participants spoke about the connections made with patients and that the stronger the connection, the greater the impact the death had on participants. Emma highlights this point when she talked about one of her favourite patients.

Usually you have your favourites who you have more of a connection with; you know a bit more about their life and their family. I’ve got a client who reminds me so much of my grandfather and I think, when he passes away, that’ll probably affect me more than other clients. (Emma)

Emma acknowledged that she had made a strong connection with this patient and because of that connection she recognised that when he passes away, the loss is likely to have a significant impact on her. Participants commonly talked about the deep sadness associated with losing a patient that they had established a connection with. Louise explained:

I happened to be reading the death notices in the paper. So I knew before his wife actually rang me. I think it just was – I felt really sad. I knew it was coming but he was a man that I actually knew a lot about his family, I knew his wife quite well, he had lots of health challenges, so I think there was a real sense of loss that I wouldn’t be seeing or treating him anymore. (Louise)

For Louise, it was the realisation that she would never see this person again. Even though she was aware that he did not have long to live, the loss still had an impact on her because she had connected with him on a personal level and she was part of his health journey.

This was echoed by Kate who talked about being involved in the regular care of a patient over a number of years. This demonstrated how emotionally invested podiatrists may become in providing quality care.

We do deal with a lot of sad things. People are always getting sick. My patients have been seeing me for 10 years. I’ve watched them go from walking spritely to all these crippling conditions. They’re getting old and sick and going into nursing homes and it is just so sad. Because they come in and they tell you their struggles and this and that. (Kate)

Kate also explained the sadness of watching older people deteriorate over a period of time when you have regularly talked about their life and personal challenges. The participants described that the uniqueness of the podiatry profession facilitated the development of connections over a long period of time that were not always understood by people outside of the profession. Fran captured the feeling of other participants when she described the emotional toll of losing a patient she was connected to.

It literally was devastating for me. I really struggled to let it go. I would be still in the grieving process and there were times when people didn’t understand. Like you know, it’s a client, how attached can you be to a client? How well can you know a client? People are not aware of how involved we are, how connected we are, and how open our heart is at times when we’re treating these people. (Fran)

In addition, some participants noted the connections they had with their patients were not always recognised by others, especially family members as can be seen by Megan’s excerpt.

And you lose these people and you don’t ever know what happened to them because I’m just the card that they stick on the fridge and the family just come in and discard all that. And often they don’t even remember, “Oh, that doesn’t matter” but I am important. I think a podiatrist plays a huge role in people’s life. It’s incredible feeling and a great responsibility, but it does burden me from time to time. (Megan)

Megan describes the frustration she feels when she is not told that one of her regular patients has passed away. Megan perceived that she played a significant role in the patient’s life and not having that connection acknowledged, devalued her importance. However, other participants had different experiences where they were unaware of just how much of an impact their contribution had made on the person. Kara indicated her surprise to the family member’s reaction towards her.

And the family comes up and hugs us after the funeral service, you realise just how much of an impact you have on their family. They consider you as part of their family. And it’s not until you get to a situation like that until you realise “Wow, they had a lot of trust in me. They treated me like I was a family member. I was a regular part of their week”. (Kara)

The participants also indicated that the family connection was often strengthened if regular contact with the podiatrist was maintained by other family members. They often talked about the challenges of talking about a deceased patient to a relative.

He won’t see any other podiatrist in the clinic. He has to only see me because I was the one who looked after his deceased wife. And whilst we’d never talk about her feet or her particular foot problems or anything really, he knows I really cared about her and there’s just this way that I know he feels closer to his wife by seeing me. (Annabelle)

Annabelle’s excerpt illustrates that a shared experience, such as a death of a patient, can easily deepen the connection made with relatives that are also patients.

### Theme 2: Willing to share and listen

There was a strong sense by participants that even though they are health professionals, death can have a significant emotional impact and it was not always acknowledged.

I think as a profession maybe we don’t deal with it so well. We accept that it happens, but I think as a practice, not so much acknowledgement of the impact that it actually has on people. Particularly when you have been seeing someone for quite a long time, quite regularly, sometimes you see them more than you see your friends, so you know a lot about them. And I don’t know that we acknowledge that very well as a profession because I think we feel like you’ve got to keep going because it is work. (Anna)

The perception from participants was that because the emotional impact was specifically relating to ‘work’ then the reaction should be different, almost hidden, compared to if it was a close family or friend. This perception was further explored by Kara, who indicated because death and grief was not always openly talked about within the workplace, there was a sense of dealing with it alone and therefore not seeking support from others.

Sometimes I think that we feel that we should grieve alone. We just kind of forget that we can use other people’s shoulders and we don’t need to grieve alone. I think just talking about feelings, someone that you trust, a family member or another person in the clinic, or a mentor – somebody. (Kara)

Brett took this idea further, acknowledging that even though everybody grieves differently, creating the awareness that it is OK to ask for help if required, it is OK to admit that the grief has had a profound impact, and that these were important strategies to start to deal with that grief.

There are not rules when it comes to grieving. Some people get over things quickly, others don’t. Some people deal with it with talking, others with ignoring. So I think that needs to be presented because some people might feel a really profound loss whereas others might kind of be stoic. So I think creating awareness is important and also giving people the opportunity for support networks. The more opportunities we have for that, the more we can share our experience in areas like grief. (Brett)

While the participants recognised the need to share their grief with someone, equally important was a supportive culture within the workplace that provided a safe environment for the practitioner to discuss sensitive topics such as the emotional impact of grief and loss.

In my team we ask for support often early, so if we’re struggling with something like that then we get supported and we talk about it and we’ll hug and we’ll cry and we’ll talk about how wonderful this person was. You have to speak to someone who has empathy, who understands. So it’s literally, speak to a colleague who’s going to know exactly what you’re going through. Having someone who can help you and support you understand what it is that you need to process. (Fran)

Fran talked about the importance of having someone to speak to who understands, who has empathy. Participants emphasised the importance of finding the right person that you felt comfortable with. This may be an individual person, which participants indicated were more likely to be colleagues, as they could appreciate the circumstances. Or some participants talked about debriefing within a team environment, as discussed by Tim.

Quite often we’ll be able to reminisce at a team meeting or a case study or have a laugh about the different bits and pieces they threw up throughout their journey. I think everyone needs to allow themselves to grieve, that there is fostered, within the workplace, an open discussion with both the individuals who may experience that, as well as the management and understand that [grief] can be varied. (Tim)

Regardless of whether the practitioner sought to debrief with an individual colleague or their team, the important component was the ability to be willing to share their grief with others and willing to listen to support those around them.

### Theme 3: Creating support through starting the conversation.

The final theme was creating support through starting the conversation. This was an area commented on by all participants. This indicates a need for greater emphasis by the podiatry profession to talk more formally about the impact death and dying has on practitioners. Participants discussed how starting the conversation as a profession was likely to increase our willingness to access support.

Start talking about death. We’re always saying “diagnose, treat, and fix”. But we also need to actually focus on the death part – how we deal with death and what it means. Because I know there would be a lot of new grads out there who may not have had family members that have passed. Some people are a little bit innocent, are blinkered. I think being aware of what you do, or who you should go to, or where you can go for help, just talking about feelings. (Kara)

Kara highlights the point that often the focus is on the diagnosis and management of patients without consideration of the emotional impact of working as a podiatrist. The perceived level of naivety among new graduates and not being adequately prepared to deal with these types of situations was also illustrated in Kate’s excerpt.

Talking about grief and loss and how you feel when a patient dies. “What are the symptoms and signs I need to look for to actually look after myself?” And that then gives them an opportunity to take ownership. That’s one thing I found when I was a new grad. There wasn’t anything that sort of prepared us for the situations. (Kate)

Not being provided with tools to recognise the significant emotional impact of a patient death has on the practitioner limits opportunities to know when and where to access support to help deal with this potentially challenging topic. In addition, participants noted that they had to learn from personal experience rather than having formalised education in this area and appreciating that grief and death was common part of the podiatry profession. As James explains, participants felt it was important to encourage, especially younger podiatrists, to speak up if they were struggling so that they could receive the necessary support.

When I was studying I didn’t realise this would happen. You don’t realise at the start that you have to deal with these issues and these situations. Be open and don’t be ashamed of seeking help from family, friends, colleagues and professional services with grieving the loss of a patient. (James)

Starting the conversation early and developing strategies to deal with grief, possibly as part of student training, was a frequent discussion point made by participants. Alongside these elements, Annabelle emphasised that grief and loss were a common occurrence within the podiatry profession and that building resilience to deal with these types of emotions was an important aspect to assist podiatrists in coming to terms with their own reactions to these difficult situations.

Some tools and reference points to go to when they’re faced with grief. Opportunity to have sessions on resilience and just accepting that there is a fair bit of grief with the job. (Annabelle)

## Discussion

This research suggests there was a profound impact that the death of a long term patient had on the podiatrist, especially when a deep connection had been established. The concept is not new or unique and there may be varying degrees of grief depending on the unique bond a health professional has with a patient [28]. The nature and scope of podiatrists’ practice provides the opportunity to develop close long lasting relationships with their patients. As such the resultant emotional investment within the patients’ healthcare journey can lead to a range of grief responses [29].

One challenge identified by participants was the perception that work related grief was a silent matter and its impact often remained hidden from those around them. This belief, which has been referred to in other health professions as “private shadows” [28], may be perpetuated by the lack of open discussion on the topic from within the profession. Professional culture can often facilitate a stigma associated with expressions of grief by healthcare providers which feeds this concept of isolation in sadness [30]. Therefore, some health professionals may be reluctant to acknowledge the personal impact of the grief, especially if this area is not openly talked about.

It was common for participants to talk about the need for debriefing in person as an important coping strategy to deal with the emotional impact. They described this person as usually a colleague who knew the particular patient and they felt comfortable opening up to. Debriefing is seen as a critical support mechanism during emotional events for healthcare providers to vent emotions and move through the grief cycle [31]. This is commonly provided, encouraged and at times, mandatory for health care providers in the public health care setting. However, isolated settings where a solo practitioner practice required the podiatrist to proactively seek assistance. If podiatrists are not openly discussing the emotional impact of patient death, this may make it challenging for some practitioners, especially new graduates to open up about their feelings of grief, even to colleagues. Grief can also be linked to professional burnout, especially if practitioners do not have the necessary skills to recognise and manage their grief [29, 32, 33]. Professional burnout is not new to podiatry and can be triggered by a number of factors [8]. Acknowledging the impact of work-related grief and developing effective coping strategies may reduce the risk of some podiatrists developing professional burnout.

One of the most significant findings from this study was that all participants indicated that they had no formal training on how to deal with the psychological impact associated with the death of a long term patient. This lack of knowledge limited their ability to initiate management strategies for coping with the emotional stress relating to patient death. The ability to recognise potential triggers to emotions such as grief is seen as important in targeting interventions to build resilience [29]. Profession specific and timely education about death and dying should be considered, but the need for such is often reactive [29, 34]. However, it is recognised that across healthcare professions more education is required to better support practitioners in this area [31, 35]. Limited opportunities to undertake education on understanding and managing grief, especially for new graduates was evident and the development of resources targeting this area is warranted within the podiatry profession. Further research is required to determine the most effective approaches to support practitioners and whether education strategies should be specifically targeted at undergraduate programs, through professional associations or a combination of both.

## Limitations

It is important to acknowledge the self-selection bias of participants within this research and this limitation was considered during the planning stage. There was potential that podiatrists who responded to advertising and chose to participate in the study may have had extreme responses and experiences relating to patient death. This would thereby impact the transferability of the results to the broader profession. Interestingly, podiatrists who participated had variable responses to grief, some describing minimal self-impact however noted that their colleagues were at times impacted. They also discussed their varied beliefs on grief-related education, identified a need for ongoing professional development from professional associations or wished to discuss grief and how the connection with their community or religious beliefs assisted following the death of a patient.

Due to the participants being geographically dispersed across Australia, it was not feasible to conduct face to face interviews with participants. By undertaking telephone interviews data analysis may have been impacted as non-verbal cues such as facial impressions were not considered during data collection. The use of telephone interviews may make it harder to develop a rapport with the participant [36]. However, there is evidence that suggests that when dealing with sensitive topics participants may feel more relaxed if the researcher is not present in the room [37, 38]. Both the BRS and aMBI were undertaken as exclusion criteria. No participant who consented to being in the study demonstrated extreme results on these scales. In hindsight, these may have been an unnecessary inclusion within the study design.

## Conclusion

This is the first known research providing an insight into the impact of a long term patient’s death on podiatrists. The findings indicate that death and dying can be an emotive topic and one which podiatrists may not be prepared for, yet likely to have to deal with throughout their career. The different levels of emotional investment the podiatrist placed in the patient relationship, resulted in grief manifesting in different ways. Grief can be deeply personal and personal or professional debriefing was strongly highlighted as a way to move through the grieving process. However, limited opportunities currently exist to appropriately prepare practitioners to deal with this emotional impact. As such the development of specific educational resources targeting this area are warranted to better support practitioners over their professional careers.

## Abbreviations

AHPRA: Australian Health Practitioner Regulation Agency
aMBI: Abbreviated Maslach Burnout Inventory
BRS: Brief Resilience Scale
IPA: Interpretative phenomenological analysis
SD: Standard deviation
